# Supplementary material for: Bipolar disorder among married women in Bangladesh: Survey in Rajshahi city
Source: PLoS One. 2020 Feb 27;15(2):e0229539. doi: 10.1371/journal.pone.0229539 (PMC7046238; doi:10.1371/journal.pone.0229539)
Supplement: Attachment — Submitted filename: Response to Editor and Reviewers.doc [file pone.0229539.s003.doc]

**Response to Editor and Reviewers**

**Journal Name:** PLOS ONE

**Tracking No. (Manuscript ID):** PONE-D-19-24745R1

**Manuscript Title:** “Bipolar disorder among married women in Bangladesh: Survey in Rajshahi city"

Dear Editor,

Thank you very much for providing your comments on our manuscript. We have modified and revised the manuscript accordingly, and detailed corrections point-by-point is given below:

**Comment:**

The authors have incorporated most of the comments in the revised manuscript; however, some of the writing is a bit imprecise, the manuscript be edited by a professional English language editor before publication.
**Author’s response:** Thank you very much for your useful comments on our manuscript.

(i) According to your suggestion, we have tried to precise our manuscript.

(ii) As you suggested, we have sent our manuscript to Professor Jyoti Sarkar, Department of Mathematical Sciences, School of Science, Indiana University Purdue University Indianapolis for improvement of language in this manuscript. He has revised English throughout the manuscript.

We would like to thank the editor and reviewers for the valuable comments. We have revised the documents to the best of our ability, but we will definitely be happy to provide further improvement if there are further clarifications required.

With best regards

Dr. Md. Golam Hossain

Professor of Health Research Group

Department of Statistics, University of Rajshahi

Rajshahi-6205, Bangladesh

E-mail: hossain95@yahoo.com
